# Return to baseline arsenic concentrations after 1 year on gluten‐free diet in children with celiac disease: A prospective cohort study

**DOI:** 10.1002/jpr3.70135

**Published:** 2025-12-26

**Authors:** Nan Du, Clara Baek, Rachel Rosen, Tracy Punshon, Jocelyn A. Silvester

**Affiliations:** ^1^ Division of Gastroenterology, Hepatology and Nutrition Boston Children's Hospital Boston Massachusetts USA; ^2^ Department of Biological Sciences Dartmouth College Hanover New Hampshire USA; ^3^ Celiac Center, Division of Gastroenterology Beth Israel Deaconess Medical Center Boston Massachusetts USA

**Keywords:** environmental contaminants, heavy metals, medically prescribed diet

## Abstract

**Objectives:**

Lifelong adherence to a gluten‐free diet (GFD) is the primary treatment for celiac disease (CeD). Concerns have been raised about increased exposure to contaminants in a GFD because rice, which naturally bioaccumulates arsenic and other environmental contaminants, is commonly used as a substitute for gluten. In this study, we aimed to determine whether elevated urinary arsenic and other heavy metal concentrations, previously observed after 6 months on a GFD, persisted after 12 months on a GFD.

**Methods:**

Single‐center prospective longitudinal cohort study of children (age 2–18 years) with biopsy‐confirmed CeD who initiated a GFD between January and May 2022. The primary outcome was the change in urinary arsenic concentration between baseline diagnostic endoscopy and 12 months on a GFD.

**Results:**

Twenty‐one of the 35 (60%) participants provided a follow‐up sample. After 12 months on a GFD, total median urinary arsenic concentration returned to baseline (3.42 vs. 4.93 μg/L, *p* = 0.17). The median total nail arsenic collection did not differ significantly between endoscopy and 12 months on a GFD samples (*p* = 0.56). Only urinary molybdenum concentration was increased between endoscopy and 12 months of a GFD.

**Conclusions:**

Children with CeD may have increased arsenic exposure as they transition to a GFD, but concentrations return to baseline within 1 year. Future studies are needed to identify dietary contributors and guide strategies to limit excess arsenic exposure.

## Abstract (highlights)

**What is Known:**

- Lifelong adherence to a gluten‐free diet (GFD) is the primary treatment for celiac disease.
- Rice, a commonly used gluten‐free grain, naturally bioaccumulates arsenic and other heavy metals.
- After 6 months of a GFD, children with celiac disease were exposed to 3–4 times more arsenic than when they were on a gluten‐containing diet.

**What is New:**

- Although GFDs are a risk factor for environmental heavy metal exposure, elevated urinary arsenic levels observed at 6 months normalized by 12 months on a GFD.
- Toenail analysis over 12 months on a GFD showed a nonsignificant trend toward higher chronic arsenic exposure.

## 1 INTRODUCTION

Celiac disease (CeD) is a systemic disorder that affects 1%–3% of Americans, with immune‐mediated damage to the intestinal villi driven by gluten exposure. Lifelong adherence to a gluten‐free diet (GFD) is the primary treatment for CeD. Concerns exist regarding increased exposure to arsenic and other heavy metals in a GFD because rice, which naturally bioaccumulates arsenic and other contaminants due to being grown in flooded soil, is commonly used as a substitute for gluten.^1^, ^2^ Long‐term exposure to arsenic, even at low levels, has been linked to a range of adverse health effects in different body systems, including developmental, metabolic, neurological, and cardiac.^3^, ^4^, ^5^, ^6^, ^7^ Most prior studies examined arsenic exposure from water rather than dietary sources in adults and adolescents.^8^ How these low levels of arsenic and heavy metals may impact developing infants and children is not known.

Previously, we studied a cohort of children with newly diagnosed CeD and found that they had significantly increased urinary arsenic concentrations 6 months after transitioning to a GFD.^9^ We had also identified four other heavy metals (cobalt, nickel, strontium, and barium) that had also increased concentrations in the urine after adoption of GFD. Here, we report follow‐up in participants following a GFD for longer than 12 months to see if these observations persist.

## 2 METHODS

### 2.1 Ethics statement

The Boston Children's Hospital Institutional Review Board approved the study protocol. Informed consent by participants was sought and documented.

### 2.2 Study population

Previously, we reported on a prospective cohort of 50 children (between ages of 2–18 years) diagnosed with CeD between January and May 2022.^9^ At the time of endoscopy, participants provided urine and toenail samples along with a 3‐day dietary record. In this cohort, urinary arsenic concentrations were significantly increased from baseline after 6 months of a GFD. For the current study, the 35 participants who had provided samples after 6 months on a GFD were asked to submit additional urine and toenail samples after 12 months on a GFD and complete a survey about symptoms, GFD adherence, and tobacco smoke exposure, as this contains arsenic and other heavy metals.

### 2.3 Urine concentrations for arsenic and other heavy metals

The primary outcome was the change in urinary arsenic concentrations between the esophagogastroduodenoscopy (EGD) and 12 months after GFD initiation. Secondary outcomes included comparing the urinary arsenic concentrations between 6 months and 12 months after GFD initiation as well as changes in other urinary heavy metal concentrations. Spot urine samples collected 12 months after GFD initiation were aliquoted into 1.8 mL vials within 24–72 h then stored at −80° C.^10^ Urinary concentrations of arsenic and 22 other elements (Be, Al, V, Cr, Mn, Fe, Co, Ni, Cu, Zn, Se, Sr, Mo, Ag, Cd, Sn, Sb, Ba, Hg, Ti, Pb, and U) were determined at the trace element analysis (TEA) Core at Dartmouth College using the reference standard for urinary element analysis^11^‐inductively coupled plasma mass spectrometry (Agilent 8900 ICP‐MS; Agilent Technologies) interfaced with an Agilent liquid chromatograph 1260 equipped with Thermo AS7, 2 × 250 mm column and a Thermo AG7, 2 × 50 guard column.^12^ Seronorm Concentration 1 and 2 urine and UTAK laboratory quality certified urine standards were run after each calibration and every 20 samples.

### 2.4 Dietary arsenic estimation

Dietary arsenic exposure was estimated using a 3‐day dietary record of rice and non‐rice‐based foods that commonly contain arsenic, including seafood (tuna, dark meat fish), fruits (apple, pear, and grapes), and beans. Servings were defined for each category (fruit and juice, vegetable and beans, meat and fish, cold/hot cereals not prepared with home tap water, granola or snack bars, and rice‐based products) and can be found in Data S1. If any of the products contained rice in the ingredient list, they were recoded as rice‐based products. The food diaries were collected on paper during clinic visits or returned via postal mail using the same protocol utilized previously in our prospective cohort, which had been influenced by the New Hampshire Birth Cohort Study.^13^ Urine was collected on Day 4.

### 2.5 Nail arsenic and other heavy metals

Toenail samples were collected as a biomarker of long‐term (6–12 months) arsenic exposure since arsenic binds to keratin. Toenail sample collection was performed as previously described with a stainless‐steel nail clipper.^9^ Toenail arsenic values were not used as the primary outcome measure given the lack of robust normative data for the US population, but we were able to compare to prior samples measured on the same patients.^14^

### 2.6 Statistical analysis

Assuming a power of 80% and *α* of 0.05, a sample size of 15 was needed to complete this analysis. Standard descriptive summaries were used to summarize data. Paired Wilcoxon signed rank test, analysis of variance, Spearman correlation, and multivariate regression were performed in the R computing environment version 2023.03.0+386^15^ in RStudio.^16^ *p* Values less than 0.05 were considered statistically significant. When making multiple comparisons (as with other urinary heavy metals), we applied a conservative Bonferroni correction.

## 3 RESULTS

Of the 35 participants who provided a urine sample after 6 months on a GFD, 21 (60%) provided a follow‐up urine sample, and 20 provided a toenail sample after 12 months on a GFD. There was no significant difference in age (*p* = 0.52), sex (*p* = 0.33), or race (*p* = 0.66) between the study participants and those who did not submit a follow‐up sample after 12 months on a GFD.

### 3.1 Demographics

The study population (48% female) primarily self‐identified as white (90%), which reflects the racial composition of the CeD clinical population. All participants resided in nonsmoking households, while the majority (62%) had an annual household income >$100,000 and at least one parent with a college degree (96%). More than half (52%) had a family history of a relative with CeD. There were 15 participants with a repeat transglutaminase immunoglobulin A (TTG IgA) on a GFD, which was obtained after 12 months on a GFD (mean value of TTG IgA was 7.4, with normal TTG IgA defined as <10); 13 (87%) were within normal limits, and the other two had decreased substantially.

### 3.2 Dietary analysis

All participants were following a GFD with 90% (19/21) stating they follow either a strict GFD or usually GFD with a rare unintentional gluten consumption. The 72‐h dietary records confirmed that there was no recorded gluten ingestion. Eighty‐six percent of the participants consumed tap water. The most common non‐rice sources of dietary arsenic were chicken, apples, and brussels sprouts/broccoli/cauliflower. Common rice‐containing sources of arsenic were gluten‐free pasta, rice, and cereal bars. At baseline (i.e., prior to diagnosis), these participants consumed an average of 3.8 servings per day of non‐rice sources of dietary arsenic and 3.4 servings of rice‐containing sources of dietary arsenic per day. After 6 months of a GFD, consumption of rice‐containing sources of dietary arsenic increased significantly (8.8 servings, *p* < 0.001) whereas there was no significant change in servings of non‐rice sources of dietary arsenic (4.5 servings, *p* = 0.46). After 12 months on a GFD, consumption of non‐rice‐containing (4.6 servings per day, *p* = 0.97) and rice‐containing (9.3 servings per day, *p* < 0.001) sources of dietary arsenic remained increased from baseline. Rice‐containing servings were not associated with family history, alternative non‐rice sources of dietary arsenic, marital status, parental educational level, or parental income (*p* = 0.64).

### 3.3 Urinary arsenic concentrations

**Figure 1:**
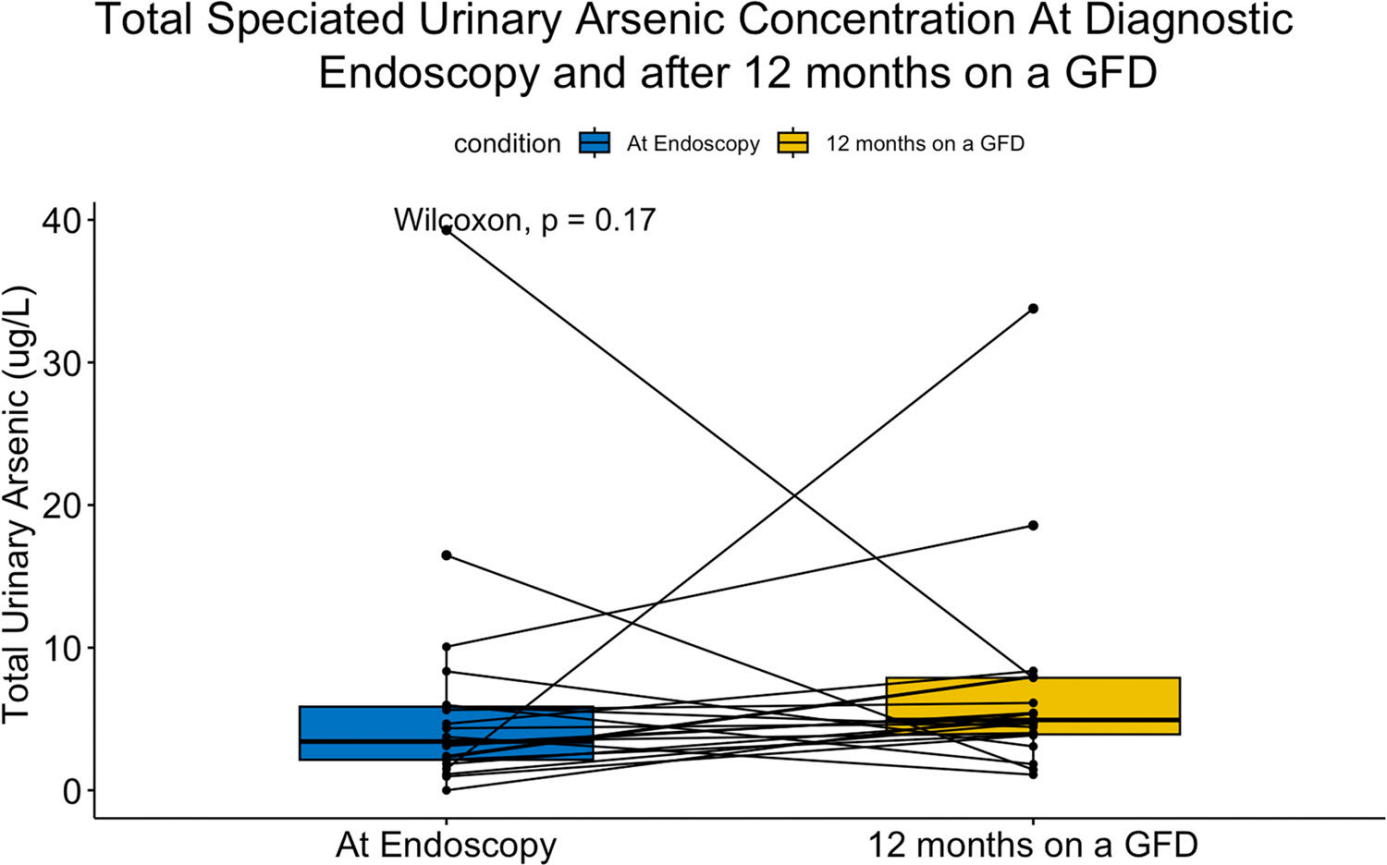
Total speciated urinary arsenic concentrations at diagnostic endoscopy and after 12 months of a GFD. The speciated arsenic concentrations refer to the sum of inorganic arsenic, monomethylarsonic acid, and dimethylarsinic acid. GFD, gluten‐free diet.

Median measured total urinary arsenic concentration returned to baseline after 12 months on a GFD (3.42 vs. 4.93 μg/L, *p* = 0.17; Figure 1), and the maximum was 34 μg/L, which is well below the threshold for arsenic toxicity in urine(100 μg/L) established by the Agency for Toxic Substances and Disease Registry in the US Department of Health and Human Services.^17^ Household income was inversely correlated with urinary arsenic level after 12 months on a GFD (0.008). For comparison, median measured total urinary arsenic concentration at 6 months was 12.8 μg/L, which was increased compared to baseline (*p* = 0.008) and 12 months on a GFD (*p* = 0.049) (Figure S1).

### 3.4 Other urinary heavy metal concentrations

**Figure 2:**
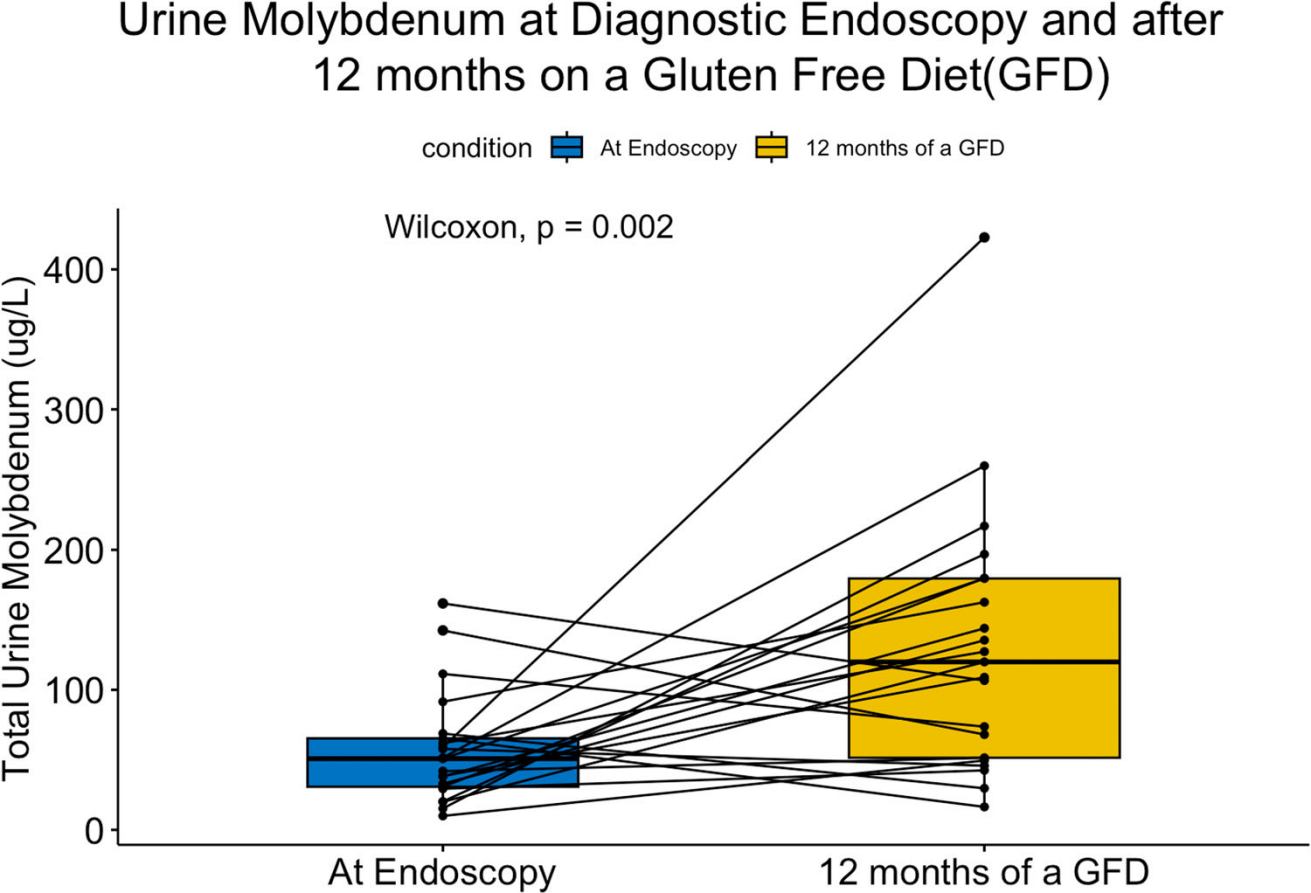
Total urine Mo concentrations at diagnostic endoscopy and after 12 months of a GFD. GFD, gluten‐free diet; Mo, molybdenum.

Current evidence supports using urine molybdenum (Mo), tin, cadmium, mercury, and uranium concentrations as a biomarker of dietary exposure.^18^ Only urinary Mo concentration was possibly significantly increased between endoscopy and 12 months of GFD (*p* = 0.002, Bonferroni corrected *p* < 0.0022; Figure 2).

### 3.5 Heavy metal concentrations in toenails

**Figure 3:**
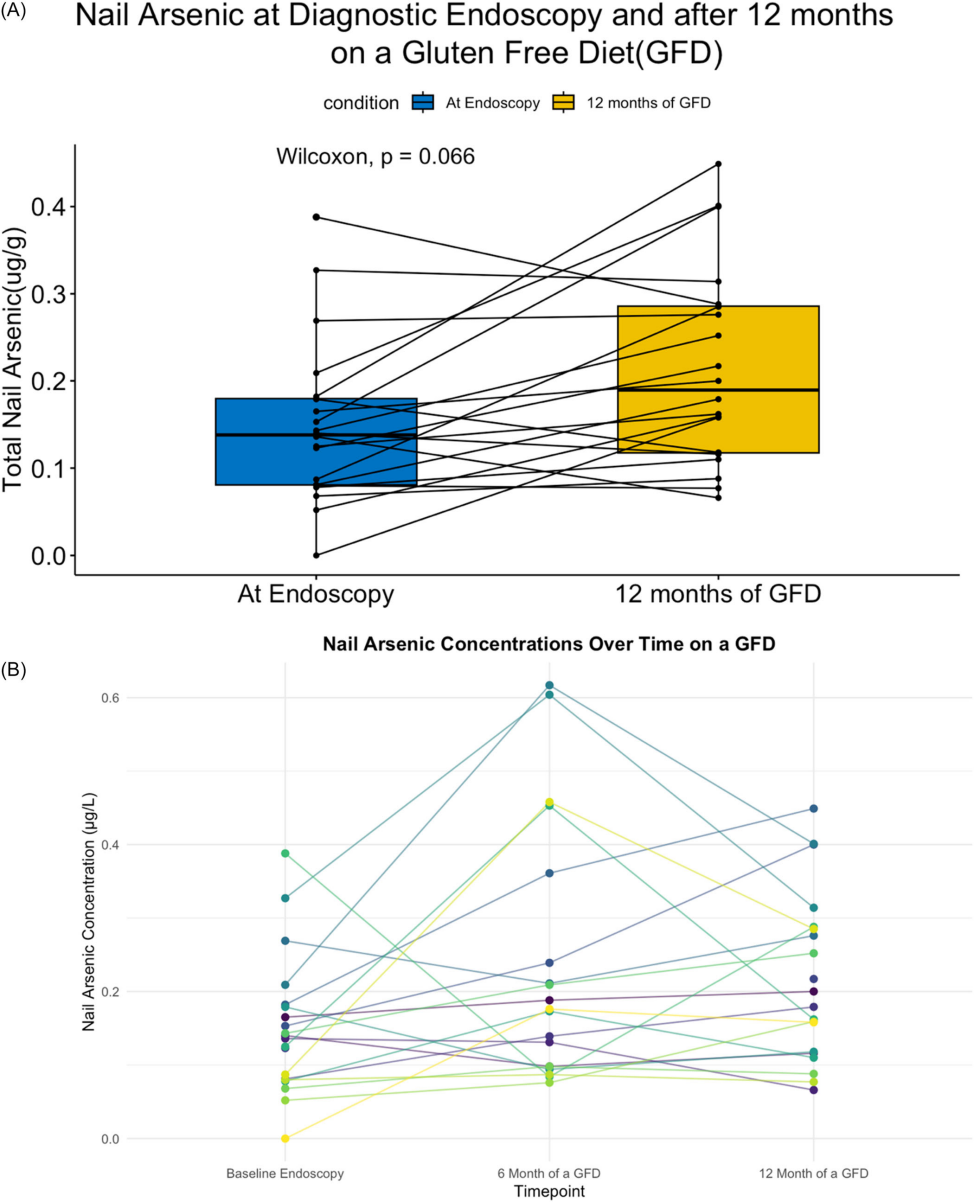
Total nail arsenic concentrations at diagnostic endoscopy, after 6 months of a GFD, and after 12 months of a GFD. (A) Total nail arsenic concentrations at diagnostic endoscopy and after 12 months of a GFD. No significant difference between the two time points. (B) Total nail arsenic concentration trends over 1 year on a GFD. GFD, gluten‐free diet.

The median total nail arsenic collection did not differ significantly between endoscopy, after 6 and 12 months on a GFD samples(*p* = 0.565) (Figure 3A,B). This lack of a significant difference may be attributed to the smaller sample size for the follow‐up collection, which may have lacked sufficient statistical power to be able to assess differences in toenail arsenic concentrations.

**Figure 4:**
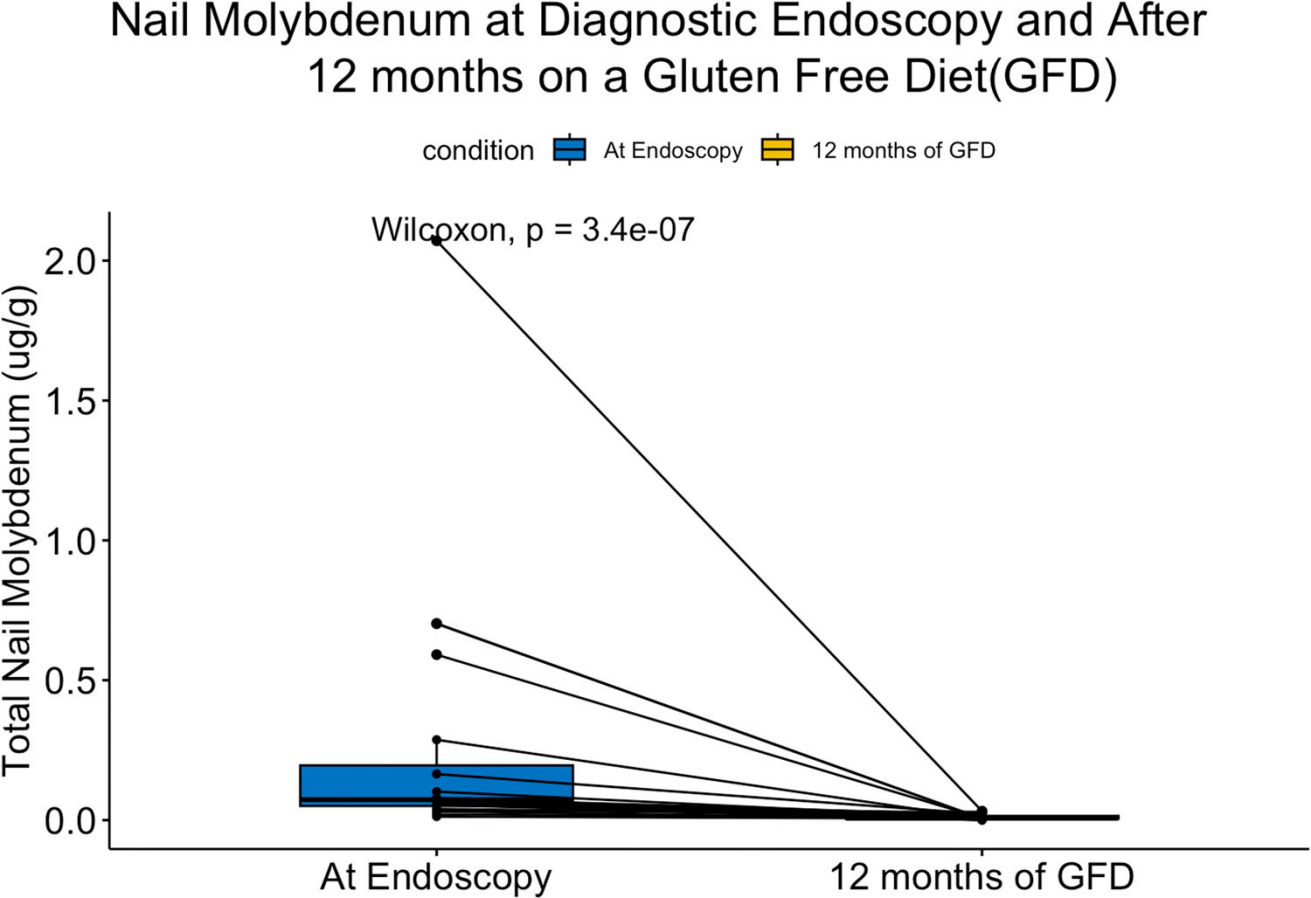
Total nail molybdenum concentrations at diagnostic endoscopy and after 12 months of a GFD. GFD, gluten‐free diet.

Current evidence supports using toenail cobalt, copper, iron, manganese, Mo, vanadium, selenium, zinc, titanium, mercury, cadmium, chromium, aluminum, nickel, beryllium, and uranium concentrations as a biomarker for exposure.^19^, ^20^ After applying a conservative Bonferroni correction for multiple comparisons, only Mo and titanium concentrations were statistically significantly different between endoscopy and after 12 months of a GFD (*p* < 0.0022). Of these, only the toenail concentrations of Mo were likely to be clinically meaningful due to the very low levels of toenail titanium (Figure 4). While iron concentrations did show an overall increasing trend in toenails, unfortunately, toenail iron is not a proxy for serum iron biomarkers.^21^

## 4 DISCUSSION

In this prospective longitudinal cohort, urinary arsenic concentrations were higher at 6 months following transition to a GFD, but returned to baseline levels at 12 months. After 12 months on a GFD, median urinary arsenic concentrations were also comparable to population reference data (National Health and Nutrition Examination Survey [NHANES]).^22^ This finding is particularly important as it offers reassurance that prolonged adherence to a GFD, even one that may initially contain higher arsenic concentrations from rice‐based food consumption, does not necessarily result in persistent increased arsenic exposure or increasing arsenic accumulation over time. There are many potential health risks of moderately elevated total urinary arsenic, including increased cardiometabolic risk, alterations in executive functioning, and neurodevelopmental delays.^5^, ^8^ Continued vigilance is warranted; however, while short‐term urinary arsenic levels returned to baseline, chronic exposure assessed via toenails as a biomarker increased, but the difference was not statistically significant. This reflects limited statistical power due to the smaller follow‐up sample size.

We observed decreased urinary arsenic excretion even though participants did not change their consumption of rice and increased their servings of other dietary sources of arsenic between 6 and 12 months on a GFD. This may reflect differences in arsenic absorption related to decreased intestinal permeability as the mucosa heals on a GFD.^23^ This argument is bolstered by the self‐reported excellent GFD adherence and normalization or near normalization of TTG IgA within 1 year of starting a GFD in this cohort. Animal data have been mixed, with some studies showing reduced permeability lowers absorption, while others suggest younger mice with healthier mucosa have greater arsenic uptake.^24^ Alternatively, many highly processed gluten‐free foods contain “organic brown rice syrup,” which is particularly rich in arsenic. It may be that this is not captured by standard dietary surveys and/or that families shift towards more natural/less processed rice‐containing foods as the impact of the higher cost of gluten‐free processed foods takes its toll on the family budget. Another potential hypothesis is that, while the primary purpose of food processing is not to remove arsenic or other heavy metals, the processing techniques may incidentally reduce arsenic and other contaminants in ultra‐processed foods. More studies are needed to understand these dynamics so that appropriate interventions can be designed to help mitigate the effect.

Mo is an essential micronutrient present in legumes, vegetables, and animal organs.^25^ Urinary Mo concentration is considered a useful biomarker of exposure and is sensitive to changes in dietary Mo intake.^26^, ^27^ The mean urinary Mo concentrations in our study after 12 months of a GFD were significantly elevated compared to baseline (130.4 vs. 57.1 μg/L). Baseline levels were comparable to mean urinary Mo level of participants aged 3–5 years old in the 2015–2016 NHANES survey (51.6 μg/L).^28^, ^29^ There is limited data on the human toxicity of Mo, given that it is rapidly excreted in urine. Although there is a case report of gout‐like syndrome and pneumoconiosis with excessive persistent ingestion of Mo,^30^ Mo toxicity is rare, which likely reflects the efficiency of renal excretion. Curiously, while urinary Mo excretion increased, concentrations of Mo in toenails (chronic exposure measurement) decreased from 0.073 μg/g at endoscopy to 0.007 μg/g after 12 months on a GFD (*p* = 0.002). Long‐term occupational exposure to Mo (not dietary exposure) has been linked to an elevated incidence of cardiovascular disease.^31^ Given that a GFD is lifelong, further research on Mo levels in this population may be warranted. Similar to arsenic, differences in Mo concentrations may indicate subtle nutritional changes in short‐term and long‐term consumption of the GFD.

The aim of our study was to evaluate whether elevated urinary arsenic concentrations in children with CeD persisted after 12 months of a GFD. Longitudinal studies with medium and long‐term outcomes are critical for chronic diseases such as CeD that are treated with a lifelong diet restriction. While we do have both 6‐ and 12‐month data, attrition was high with only 42% of those recruited at endoscopy providing a sample at 12 months, which may have introduced retention bias. Further studies of larger cohorts with a longer follow‐up period will be crucial to confirm the pilot findings we have identified, as we were only able to assess three timepoints. Another limitation is that our study population was recruited from a single center, with each participant's baseline urinary arsenic measurement prior to GFD initiation serving as a reference for interpreting follow‐up data. Although GFDs are inherently variable, all participants had attended a standardized GFD education class after their CeD diagnosis. We acknowledge that although food diaries were collected, we could not calculate absolute arsenic exposure, and serving counts may have misestimated intake. Notwithstanding these limitations, our study was sufficiently powered to demonstrate that while there may be a transient increase in arsenic exposure on initiating a GFD, this seems to resolve by 1 year.

## 5 CONCLUSION

Although GFDs are a risk factor for environmental heavy metal exposure, the effect diminishes after 12 months of a GFD. This should provide solace to patients and health care providers alike. Future studies are needed to elucidate the mechanisms and causes of fluctuations in arsenic exposure and explore prevention and mitigation strategies. Above all, given the myriad of GFDs, nuances are important, and risk assessment should be individualized.

## CONFLICT OF INTEREST STATEMENT

The authors declare no conflicts of interest.

## Supporting information

Supplemental Figure 1: Total Urinary Arsenic Concentration Trends over 1 year on a Gluten‐Free Diet by Individual Participant.

Supplemental Method 1: Three‐Day Dietary Record of Arsenic‐Containing Rice and Non‐Rice Foods, adapted from the New Hampshire Birth Cohort.

## Data Availability

Individual participant data will not be shared. Proposals for access to study protocol and analytic code should be sent to nan.du@childrens.harvard.edu.
