# Supplementary figures and images for: Return to baseline arsenic concentrations after 1 year on gluten‐free diet in children with celiac disease: A prospective cohort study
Source: JPGN Rep. 2025 Dec 26;7(2):315–22. doi: 10.1002/jpr3.70135 (PMC13151007; doi:10.1002/jpr3.70135)

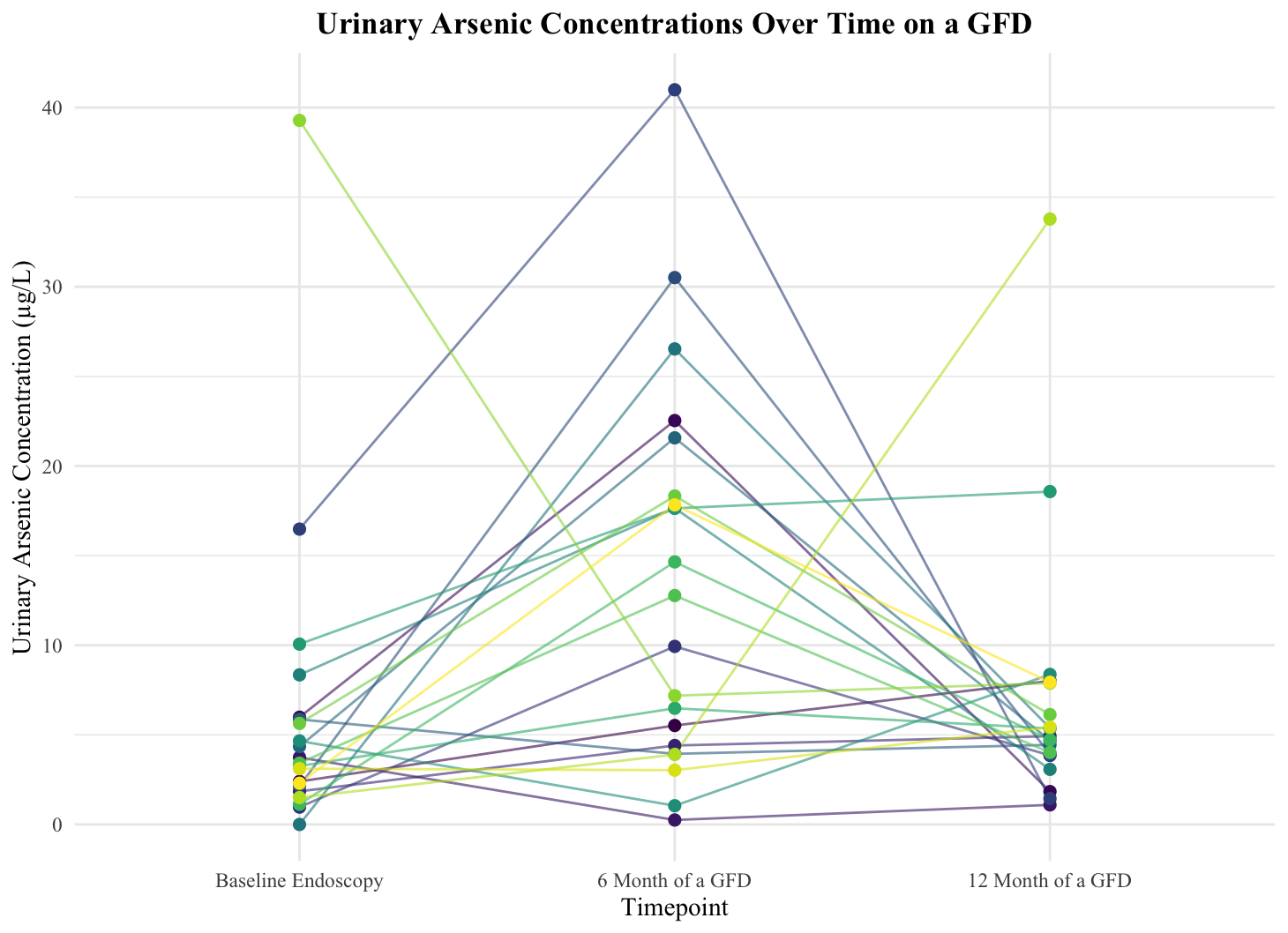

Supplement: Supplementary file 1 — Supplemental Figure 1: Total Urinary Arsenic Concentration Trends over 1 year on a Gluten‐Free Diet by Individual Participant. [file JPR3-7-315-s001.tiff]
