# Supplementary material for: Return to baseline arsenic concentrations after 1 year on gluten‐free diet in children with celiac disease: A prospective cohort study
Source: JPGN Rep. 2025 Dec 26;7(2):315–22. doi: 10.1002/jpr3.70135 (PMC13151007; doi:10.1002/jpr3.70135)
Supplement: Supplementary file 2 — Supplemental Method 1: Three‐Day Dietary Record of Arsenic‐Containing Rice and Non‐Rice Foods, adapted from the New Hampshire Birth Cohort. [file JPR3-7-315-s002.docx]

| **Food Item** | **2 Days Before Urine Collection** | **Day Before Urine Collection** | **Day of Urine Collection** | **Organic?**  ***Check box if some ingredients were organic*** | **Filtered water?**  ***Check box if home tap water was filtered*** |
| --- | --- | --- | --- | --- | --- |
|  | **DATE:** | **DATE:** | **DATE:** |  |  |
|  | Record the total number of **servings** consumed each day | | |  |  |
| **Beverages**  (One Serving = 8 ounces) | | | | | |
| Home tap water |  |  |  | ☐ | ☐ |
| Tap water at school or day care |  |  |  | ☐ | ☐ |
| Tap water at a restaurant |  |  |  | ☐ | ☐ |
| Bottled water  *What brand? _____________________________* |  |  |  | ☐ |  |
| Water from local spring  *From what spring or town?___________________* |  |  |  | ☐ |  |
| Beverages prepared with home tap water, e.g., lemonade, juice, coffee, tea, and carbonated water  *List type of beverage* |  |  |  |  |  |
|  |  |  |  | ☐ | ☐ |
|  |  |  |  | ☐ | ☐ |
|  |  |  |  | ☐ | ☐ |
|  |  |  |  | ☐ | ☐ |
| **Foods made with Home Tap Water**  e.g. oatmeal, rice cereal, grits, soup, rice, pasta, Jello, dried beans  One Serving = ½ cup prepared | | | | | |
| *List each specific food, including brand and product name, and note amount of water used if not prepared following package directions.* |  |  |  |  |  |
|  |  |  |  | ☐ | ☐ |
|  |  |  |  | ☐ | ☐ |
|  |  |  |  | ☐ | ☐ |
|  |  |  |  | ☐ | ☐ |
|  |  |  |  | ☐ | ☐ |
|  |  |  |  | ☐ | ☐ |
| **Fruit & Juice** | | | | | |
| Apple (1 apple), applesauce (1/2 cup), or apple juice (4 ounces) |  |  |  | ☐ |  |
| Pear (1 pear) |  |  |  | ☐ |  |
| Grapes (1/2 cup), grape juice (4 oz) |  |  |  | ☐ |  |
| **Vegetables & Beans** | | | | | |
| Brussels sprouts, cauliflower, cabbage, bok choy, or broccoli (1/2 cup) |  |  |  | ☐ |  |
| Canned beans; pinto, kidney, black (¼ cup) |  |  |  | ☐ |  |
| Seaweed/algae (e.g., in sushi)  *Indicate food(s) and serving size(s)?*  *Example: ¼ cup seaweed salad; 2”x3” snack sheet; 1 sushi roll* |  |  |  | ☐ |  |
|  |  |  |  |  |  |
|  |  |  |  |  |  |
| **Meat and Fish** (One Serving = 1-3 ounces) | | | | | |
| Canned tuna fish |  |  |  | ☐ |  |
| Dark meat fish: tuna steaks, mackerel, salmon, sardines, bluefish, swordfish |  |  |  | ☐ |  |
| Another other fish: e.g., haddock, flounder, sea bass, trout, bluegill |  |  |  | ☐ |  |
| Chicken |  |  |  | ☐ |  |
| **Cold or hot cereals not prepared with home tap water (not recorded on p. 1)** | | | | | |
| *List each type of cereal below, including brand and product name if possible, and # servings of each item at right (1/2 cup)* |  |  |  |  |  |
|  |  |  |  | ☐ |  |
|  |  |  |  | ☐ |  |
|  |  |  |  | ☐ |  |
| **Granola or Snack Bars** (One Serving = 1 bar) | | | | | |
| *List each type of bar below, including brand and product name if possible, and # servings of each item at right (1 bar)* |  |  |  |  |  |
|  |  |  |  | ☐ |  |
|  |  |  |  | ☐ |  |
|  |  |  |  | ☐ |  |
|  |  |  |  | ☐ |  |
|  |  |  |  |  |  |
| **Rice-based products** (One Serving = 1 item or ½ cup prepared) | | | | | |
| For example:  rice cakes, rice bread, gluten-free bread  *or*  rice milk, rice pudding, fried rice, rice from restaurants, gluten-free pasta  *List items below, including brand and product name, and # servings of each item at right* |  |  |  |  |  |
|  |  |  |  | ☐ |  |
|  |  |  |  | ☐ |  |
|  |  |  |  | ☐ |  |
| **Other** (One Serving = 1 item or ½ cup prepared) | | | | | |
| *List any food products that may have been missed above.* |  |  |  |  |  |
